# Nail dysplasia and digital hypoplasia ‒ Coffin-Siris syndrome^☆^

**DOI:** 10.1016/j.abd.2022.11.010

**Published:** 2024-06-13

**Authors:** Alba Navarro-Bielsa, Daniel Ruiz Ruiz-de-Larramendiz, Pilar Abenia-Usón, Tamara Gracia-Cazaña, Yolanda Gilaberte

**Affiliations:** a Dermatology Service, Miguel Servet University Hospital, Zaragoza, Spain; b Pediatric Service, Miguel Servet University Hospital, Zaragoza, Spain

Dear Editor,

Coffin-Siris syndrome is a clinical and genetically heterogeneous congenital disorder characterized by coarse facial features, intellectual disability, hypoplasia of the distal phalanges, and aplasia or hypoplasia of the nails.

A 7-month-old boy was seen by the dermatology service for a congenital nail disorder. The toddler had been diagnosed with mega cisterna magna, a permeable oval foramen, right renal hypoplasia, and slightly delayed psychomotor development with a risk of impaired cognitive development. Physical examination revealed dysplasia of all nails and anonychia or micronychia of the 3rd, 4th, and 5th toes and the 4th and 5th fingers (Fig. 1A‒B). The patient had characteristic facial features with a broad nasal bridge, wide mouth, and thick upper and lower lips.

**Fig. 1:**
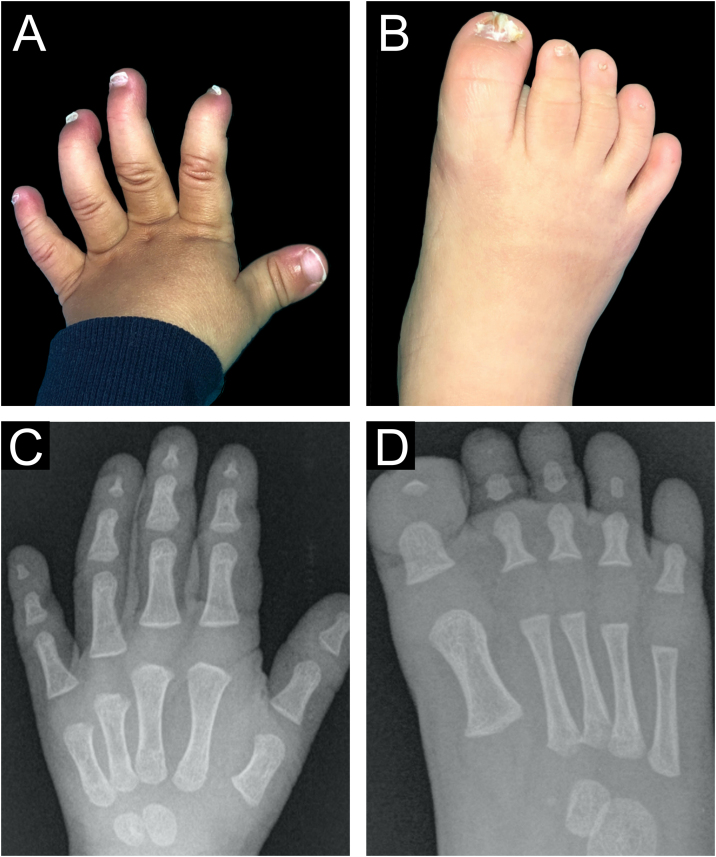
Clinical pictures with nail changes (A‒B) and radiographic (C‒D) images showing hypoplasia of the distal phalanges of the 2nd, 3rd, 4th, and 5th fingers, absent distal phalanges in the 2nd, 3rd, 4th, and 5th toes, and distal phalanx hypoplasia of the 1st toe.

Radiography of the hands and feet revealed hypoplasia of the distal phalanges of the 2nd, 3rd, 4th, and 5th fingers of both hands, absent distal phalanges on the 2nd, 3rd, 4th, and 5th toes of both feet and hypoplasia of the distal phalanx of the 1st toe on both feet (Fig. 1C‒D).

A genetic study was performed on suspicion of Coffin-Siris syndrome and revealed a heterozygous de novo mutation in ARID1A (c.2988 + 1 G > A) associated with Coffin-Siris syndrome type 2 (autosomal dominant), OMIM 614607.

Coffin-Siris syndrome is a rare congenital malformation syndrome, of which fewer than 200 cases have been described, and is caused by mutations in several genes encoding components of the BRG1/BRM Associated Factor (BAF) complex, with 12 different subtypes depending on the gene mutation, including (from highest to lowest proportion of cases) ARID1B, SMARCB, SMARCA4, ARID1A, SOX11, SMARCE1, and PHF6.^1^, ^2^ The BAF complex is an ATP-dependent chromatin remodeler and is involved in transcription, cell differentiation, and DNA repair, a phenotype-genotype correlation is emerging because mutations in BAF, have been related to abnormalities of the hair, nails and fingers. It is a clinically heterogeneous syndrome, the main signs of which include mild to severe cognitive or developmental delay, coarse facial features, and hypoplasia or aplasia of the nail and the distal phalanx of the 5th and occasionally additional fingers (toes are usually affected in individuals with multiple finger involvement). These distinctive facial features include thick eyebrows and long eyelashes, wide nasal bridge, wide mouth with thick, everted upper and lower lips, and abnormal position of the pinna. Other minor features include hypotonia, hirsutism or hypertrichosis, and sparse scalp hair, short stature, feeding difficulties, slow growth, and congenital anomalies including microcephaly, ophthalmological manifestations, and cardiac, gastrointestinal, genitourinary, and nervous system malformations.^1^, ^3^, ^4^

The differential diagnosis includes Brachymorphism-Onychodysplasia-Dysphalangism (BOD) syndrome, mosaic trisomy 9, DOORS (Deafness, Onychodystrophy, Osteodystrophy, Intellectual Disability) syndrome, fetal hydantoin/phenytoin embryopathy, fetal alcohol spectrum disorders, Mabry syndrome, Cook syndrome, Zimmermann-Laband syndrome, nail-patella syndrome, and Iso-Kikuchi syndrome. Table 1 summarizes clinical similarities and differences of these differential diagnoses with respect to Coffin Siris syndrome, the definitive diagnosis of which is genetic.^3^

**Table 1:** Differential diagnosis of Coffin-Siris syndrome.

Syndrome	Clinical features similar to Coffin-Siris syndrome	Clinical features distinct from Coffin Siris syndrome	Diagnosis
Brachymorphism-onychodysplasia-dysphalangism (BOD)	Tiny dysplastic nails, short fifth fingers, wide mouth with broad nose, mild intellectual deficits	‒	Suggested that Coffin-Siris syndrome and BOD syndrome are allelic variants
Mosaic trisomy 9	Hypoplasia of the 5^th^ digits, facial features, hirsutism, congenital cardiac, urogenital and neurologic anomalies	Skeletal anomalies and pigmentary mosaic skin lesions along Blaschko lines	Karyotype
DOORS	Hypoplastic terminal phalanges and/or nail anomalies, neurologic abnormalities, mild-to-severe intellectual disability	Deafness, osteodystrophy, and seizures	Biallelic pathogenic variants in TBC1D24. Autosomal recessive
Fetal hydantoin/phenytoin embryopathy	Small nails with hypoplasia of distal phalanges, dysmorphic facial features, digitalized thumbs, growth retardation, cognitive disabilities, cardiac anomalies	Microcephaly, ocular defects, oral clefts, umbilical and inguinal hernias, and hypospadias	History of phenytoin exposure during gestation
Fetal alcohol spectrum	Small nails, prenatal and postnatal growth retardation, dysmorphic facial features, cognitive disabilities, neurologic, urogenital, and ocular abnormalities	Musculoskeletal and auditory system abnormalities	History of fetal alcohol exposure
Mabry	Hypoplastic 5^th^digits, delayed development, coarse facial features, hypotonia, congenital heart defects	Elevated serum concentrations of alkaline phosphatase, seizures, cleft palate, megacolon, anorectal malformations	Biallelic pathogenic variants in PIGV. Autosomal recessive
Cook	Hypo/anonychia, small or absent distal phalanges and thumb digitalization	No facial dimorphism. Cook syndrome is considered a clinical form of type B brachydactyly (hypoplasia or aplasia of the terminal parts of fingers 2–5)	Mutations in ROR2 gene (9q22).
			Autosomal dominant
Zimmermann-Laband	Absence or hypoplasia of the fingernails or terminal phalanges of the hands and feet and coarse facial features.	Gingival fibromatosis	Genetic basis is unknown. Autosomal dominant inheritance has been proposed
	Hypertrichosis, cognitive disabilities		
Nail patella	Nail hypoplasia or aplasia, renal and ocular abnormalities	Patellar dysostosis, elbow dysplasia, presence of iliac horns	Mutations in the LMX1B gene.
			Autosomal dominant
Iso-Kikuchi	Anonychia or dysplasia of the nail of the index finger accompanied by underlying bone abnormalities	Rarely associated with other conditions	Genetic basis is unknown.
			Autosomal dominant inheritance has been proposed

DOORS, Deafness, Onychodystrophy, Osteodystrophy, Intellectual Disability.

The management of patients diagnosed with Coffin-Siris syndrome is symptomatic and consists of occupational, physical, and feeding therapies, including nutritional supplementation and/or gastrostomy tube placement as needed. The prognosis depends on the extent of involvement.

It will be necessary for a yearly evaluation by different specialists, like otorhinolaryngology, ophthalmology, and neurology y/o digestive to assess developmental progress and therapeutic and educational interventions.

In conclusion, Coffin-Siris syndrome is a clinically heterogeneous syndrome. While nail involvement and hypoplasia of the distal phalanges can be among the less serious clinical signs, dermatologists should be familiar with these manifestations, which are often key to establishing the diagnosis.

## Financial support

None declared.

## Authors’ contributions

Alba Navarro-Bielsa: The study concept and design; data collection, or analysis and interpretation of data; writing of the manuscript or critical review of important intellectual content; data collection, analysis and interpretation; effective participation in the research guidance; intellectual participation in the propaedeutic and/or therapeutic conduct of the studied cases; critical review of the literature; final approval of the final version of the manuscript.

Daniel Ruiz Ruiz-de-Larramendiz: The study concept and design; data collection, or analysis and interpretation of data; writing of the manuscript or critical review of important intellectual content; data collection, analysis and interpretation; effective participation in the research guidance; intellectual participation in the propaedeutic and/or therapeutic conduct of the studied cases; final approval of the final version of the manuscript.

Pilar Abenia-Usón: The study concept and design; data collection, or analysis and interpretation of data; data collection, analysis and interpretation; effective participation in the research guidance; intellectual participation in the propaedeutic and/or therapeutic conduct of the studied cases; critical review of the literature; final approval of the final version of the manuscript.

Tamara Gracia-Cazaña: The study concept and design; data collection, or analysis and interpretation of data; writing of the manuscript or critical review of important intellectual content; data collection, analysis and interpretation; effective participation in the research guidance; intellectual participation in the propaedeutic and/or therapeutic conduct of the studied cases; critical review of the literature; final approval of the final version of the manuscript.

Yolanda Gilaberte: The study concept and design; data collection, or analysis and interpretation of data; writing of the manuscript or critical review of important intellectual content; data collection, analysis and interpretation; effective participation in the research guidance; intellectual participation in the propaedeutic and/or therapeutic conduct of the studied cases; critical review of the literature; final approval of the final version of the manuscript.

## Conflicts of interest

None declared.
